# Identification and tentative removal of collagen glue in Palaeolithic worked bone objects: implications for ZooMS and radiocarbon dating

**DOI:** 10.1038/s41598-023-49242-7

**Published:** 2023-12-13

**Authors:** L. G. van der Sluis, K. McGrath, F. Thil, S. Cersoy, J.-M. Pétillon, A. Zazzo

**Affiliations:** 1 UMR 7209, Archéozoologie, Archéobotanique: Sociétés, Pratiques et Environnements (AASPE), CNRS, Muséum national d’Histoire naturelle, Paris, France; 2 Department of Evolutionary Anthropology, University of Vienna, Djerassiplatz 1, 1030 Vienna, Austria; 3 Human Evolution and Archaeological Sciences (HEAS), University of Vienna, 1030 Vienna, Austria; 4 Department of Prehistory and Institute of Environmental Science and Technology (ICTA-UAB), Universitat Autònoma de Barcelona, 08193 Cerdanyola del Vallès, Barcelona Spain; 5 Laboratoire des Sciences du Climat et de l’Environnement, LSCE/IPSL UMR 8212, CEA-CNRS-UVSQ, Université Paris Saclay, 91198 Gif-sur-Yvette, France; 6 Centre de Recherche sur la Conservation (CRC), UAR 3224, CNRS, Muséum national d’Histoire naturelle, Paris, France; 7 Travaux et Recherches Archéologiques sur les Cultures, les Espaces et les Sociétés (TRACES) UMR 5608, CNRS, Université Toulouse Jean Jaurès, Toulouse, France

**Keywords:** Stable isotope analysis, Mass spectrometry, Proteomic analysis

## Abstract

Collagen glue has been used for nearly two centuries to consolidate bone material, although its prevalence in museum collections is only now becoming visible. Identifying and removing collagen glue is crucial before the execution of any geochemical or molecular analyses. Palaeolithic bone objects from old excavations intended for radiocarbon dating were first analysed using ZooMS (Zooarchaeology by Mass Spectrometry) to identify the animal species, however peaks characteristic of both cattle and whale were discovered. Two extraction methods for ZooMS were tested to identify the authentic animal species of these objects, which revealed that these were originally whale bone objects that had been consolidated with cattle collagen glue. This is the first time animal collagen glue has been identified in archaeological remains with ZooMS, illustrating again the incredible versatility of this technique. Another technique, Fourier Transform Infrared Spectroscopy in Attenuated Total Reflectance mode (FTIR-ATR), was also tested if it could rapidly identify the presence of collagen glue in archaeological bone material, which was not the case. Two other cleaning methods were tested to remove bone glue contamination prior to radiocarbon dating, along with two modified collagen extraction methods for ZooMS. These methods were applied to bone blank samples (FmC = 0.0031 ± 0.0002, (n = 219), 47 336 ± 277 yr BP) that were experimentally consolidated with collagen glue and to the Palaeolithic bone material (ca. 15 000 and 12 000 yr BP). The experimental bone blanks produced excellent ^14^C ages, suggesting the cleaning methods were successful, however the ^14^C ages for some of the Palaeolithic material remained too young considering their contextual age, suggesting that the collagen glue contamination had most likely cross-linked to the authentic collagen molecule. More research is needed in order to gain a deeper understanding of the occurrence and elimination of cross-linked collagen-based glues in material from museum collections.

## Introduction

Before the onset of synthetic consolidants for conservation purposes, natural resins (e.g., wax, gum dammar, agar jelly, and animal glues) were commonly used^1^. Bone glue, skin/hide glue and fish glue (hereafter referred to as collagen glues) have been in use since the early nineteenth century onwards^2^ on archaeological as well as fossil remains to consolidate poorly preserved material for future generations^1,3^. However, which samples were treated with collagen glue was not always documented. Bone and skin/hide glues were usually made from cattle, and occasionally from horse^4^, rabbit or sheep^5^, although fish glue and isinglass (sturgeon swim bladder glue) were similarly used^6^. To produce collagen glue, a base or acid pretreatment is first performed on the tissue, after which the collagen is denatured into soluble gelatin through hot water extraction (hydrolytic breakdown)^5,6^. Depending on the exact treatment (temperature, pH and duration) and the original starting tissue, the final gelatin product can differ in quality and properties, such as moisture content, pH, density, ash content and viscosity^5,6^. Collagen glues are also likely to contain preservatives or additives, which are not always recorded in the production process^6^. The reversibility of collagen glues has been tested^6,7^, although this was tested only one month after application and not for any potential geochemically traceable remnants. Schellmann^8^ mentions that collagen glues show good resolubility even after centuries, unless cross-linking has occurred, which can be induced through exposure to metal salts, formaldehyde, tanning agents or unknown additives.

The presence of collagen glue in archaeological remains and palaeontological fossils has gone largely unnoticed and the extensive use of this medium is only becoming clear nowadays. This is due to the limited number of ways of identifying the presence of collagen glue, which from a chemical point of view resembles the targeted material of bone collagen that is normally used for stable isotope analysis, radiocarbon dating and palaeoproteomics. However, the presence of these glues and their impact on, for example, the radiocarbon age of some samples can be significant, as well as the subsequent archaeological implications. For example, bone glue on Neanderthal remains from Spy in Belgium resulted in radiocarbon ages that were much too young, suggesting a too recent disappearance of Neanderthals from North-western Europe^9^. The discussion that followed illustrates the obscurity of this type of contamination and its implications^10,11^. The cattle glue used on these Neanderthal remains was identified by means of DNA analysis, which is expensive and time-consuming. Bovid collagen contamination was similarly encountered in the nearly intact skull from Zlatý kůň in Czechia, affecting again the radiocarbon dates^12^. Nicholson et al.^2^ underlined the need for a simple method for collagen glue identification in fossils, after which they proposed to use the extent of racemisation of serine as a principal parameter. While undoubtedly faster and less costly than DNA analysis, a specialised laboratory still is required.

Besides its identification, collagen glue removal also needs to be addressed. There is only a single study dealing with collagen glue removal prior to radiocarbon dating, which underlines its obscurity in the radiocarbon community. Takahashi et al.^13^ tested collagen glue removal by coating a mammoth bone blank (> 50.000 year old) with a layer of hide glue (~ 1–2 mm thick) and leaving it to cure for three days. Samples were soaked in water for 24 h with periodic mixing, rinsed thoroughly and dried under vacuum before being subjected to collagen extraction. Although this treatment nearly removed the glue completely, which was sufficient for carbon and nitrogen stable isotope analysis, it was still present in small amounts in all samples. While the untreated bone blank was dated to 44 800 ± 2200 yr BP, the sample with glue was 30 050 ± 320 yr BP (1.3% glue remained). This indicates that even small amounts of collagen glue can have a significant impact on radiocarbon dating, for example, 1.3% glue remnants in bone samples of 20 000 and 10 000 years old would result in an age offset of 1650 and 445 years, respectively^13^. While removing the outer surface may help, this may not always be possible when dealing with small samples, nor be very effective on notoriously porous samples, such as whale bone. The hide glue used by Takahashi et al.^13^ was modern in age but this may not be the case for all collagen glues. Crann and Grant^14^ radiocarbon dated a range of consolidants commonly used in conservation treatments, for which they obtained modern ^14^C ages for rabbit skin glue and gelatin (hide or bone glue), while isinglass gave a ^14^C age of 585 yr BP due to the reservoir effect.

## Research aim

In this paper we aim to investigate collagen glue removal from experimental and Palaeolithic bone objects. There was no record of consolidation having been performed on the Palaeolithic objects, nor were there any visible differences in surface characteristics, however ZooMS (collagen peptide mass fingerprinting) results indicated a mixture of more than one taxon—specifically cattle and whale. As the objects originate from early twentieth century excavations, a period when the use of animal-based glues for conservation purposes was common practice, the chimeric ZooMS results suggested the likely presence of animal collagen glue. To determine whether collagen glue could be successfully removed for radiocarbon dating purposes, two cleaning methods were tested on bone blank samples that were experimentally consolidated with one of three collagen glues and artificially aged in a climate chamber. Artificial aging (here thermal) is used to reproduce in a controlled and rapid way the behaviour of samples (here archaeological bone coated with a modern animal glue) over time^15^. These contaminated blank samples were subjected to FTIR-ATR and ZooMS (when sufficient sample remained) to investigate whether collagen glue contamination could be detected with these techniques. Here we report the potential removal of collagen glue from bone objects prior to radiocarbon dating, along with the usefulness of two techniques (ZooMS, FTIR-ATR) to rapidly identify the presence of collagen glue in archaeological bone material.

## Material

For the collagen glue contamination experiment, samples from the Hollis mammoth bone blank (FmC = 0.0031 ± 0.0002, (n = 219), 47 336 ± 277 yr BP^16^), were treated with three types of collagen glue:

1. Modern (rabbit) bone glue in pellets purchased from Esprit Composite, Paris, France
2. Modern (rabbit) skin/hide glue in pellets purchased from Esprit Composite, Paris, France
3. Old glue (unknown species) found in the museum in pellets supplied by Christine Bastard, Muséum national d’Histoire naturelle, Paris, France

The experimentally consolidated samples were then subjected to two cleaning methods (see Methods section) prior to radiocarbon dating to test the efficacy of collagen glue removal before applying the protocols to the archaeological samples. The experimentally consolidated bone samples were also analysed with FTIR-ATR and with ZooMS.

Important to note is that the Hollis mammoth is relatively well preserved. However, mammoth bone, or at least some anatomical elements, can be remarkably porous as well, and has in some cases been mistaken for whale bone.

**Figure 1:**
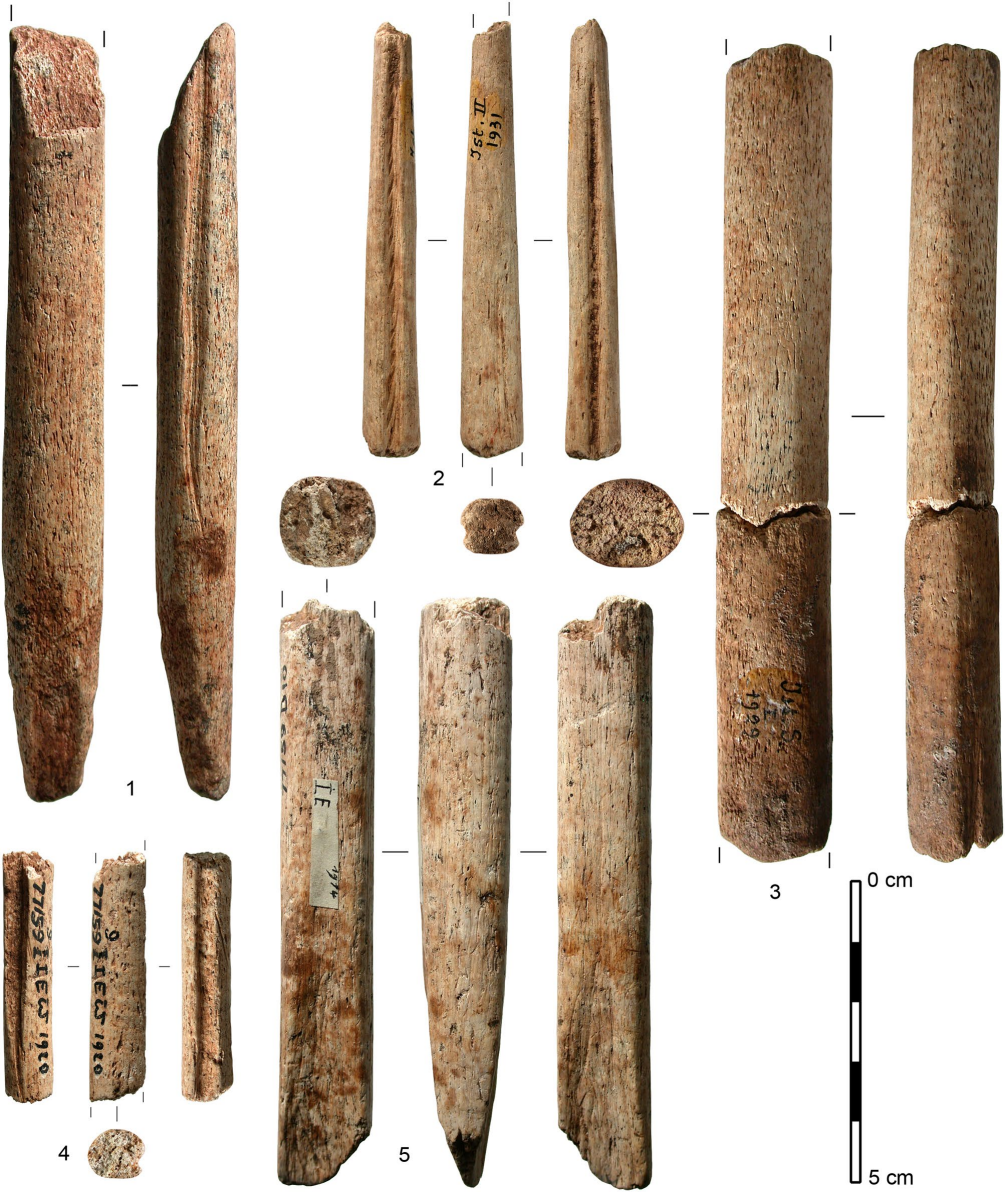
Five of the 12 fragments of objects made of whale bone analysed in this study. 1: 7 IST SI 1929 (MUSE21165), wedge; 2: 10 IST II 1931 (MUSE21166), projectile point; 3: 19 IST SI 1929 (MUSE21168), unidentified object; 4: 2 IST Ew 1920 (MUSE21164), projectile point; 5: 637 IST E 77159 D10 (MUSE21161), foreshaft.

The archaeological samples in question (Fig. 1) were excavated from the site of Isturitz, an important Upper Palaeolithic cave site in the Pyrénées-Atlantiques *département* of France. Excavations have been performed by Passemard (1912–1923), Saint-Périer (1928–1949), Turq and Normand (1996–1998), Barandiarán et al.^17^, and Normand (2000–2010), yielding material that mainly dates back to the Aurignacian, Gravettian, Solutrean and Magdalenian periods. In total, 12 archaeological bone objects originating from old excavations (1914–1937) of the Magdalenian layers, produced ZooMS peptide markers characteristic for both whale and cattle, while one additional sample (an antler sample analysed for comparison purpose) revealed peaks characteristic for both reindeer and cattle. These samples came to light as they were analysed in the framework of a larger project, called PaleoCet, in which bone objects were subjected to ZooMS and radiocarbon dating to investigate whale exploitation in prehistoric times. In an effort to remove or reduce the signal from the contaminating collagen, these 13 bone samples were subjected to two modified ZooMS extraction methods, and to the most effective cleaning method for radiocarbon dating as determined by the aforementioned contaminated Hollis bone blank experiment.

## Methods

### Experimental contamination of Hollis bone blanks with collagen glue

Collagen glue pellets (25 gr) for the three types of glue were mixed with 80 mL of tap water and heated in a hot water bath to 50 °C, as suggested by a colleague in the museum (Monsieur M. Lemoine) who is a craftsman-bookbinder by trade. The glues were already fully dissolved at 36 °C. Six bone blank samples were consolidated with each glue by submerging them entirely into the dissolved glue to represent the ‘worst case scenario’, and left to dry for three days, after which three samples were placed in a climate chamber for artificial ageing for 28 days at 50 °C with a relative humidity of 80%, while the other three were left in the laboratory to cure for 28 days at room temperature (Fig. S1 in suppl.). For the artificial ageing, the sample was suspended in a climatic chamber (oven with controlled temperature and humidity) and to obtain significant changes, a temperature higher than the ambient temperature but lower than the denaturation temperature of modern bone collagen was chosen (knowing that 50°C is generally the minimum chosen for this kind of test to produce significant changes in a few days or weeks)^15^.

The consolidated bone blank samples were subsampled (i.e. cut) to create three subsamples (in chunks) to test each of the two cleaning methods and the ABA only treatment.

### Collagen glue removal methods for radiocarbon dating

Cleaning steps were performed in double distilled (DI) water, which is slightly acidic with a pH of 5 after the uptake of CO_2_ from the air. Two cleaning methods were tested, which were the same apart from the temperature of the heated bath: the heated bath was 40 °C in cleaning method 1, while this was 50 °C in cleaning method 2. Contaminated samples were first soaked in water for 48 h on a shaker platform, after which they were subjected to a 10-min sonic bath, followed by a heated bath (method 1: 40 °C, method 2: 50 °C) for 60 min, and another 10-min sonic bath, then rinsed and air dried. The DI water was renewed between each step.

### Bone collagen extraction (ABA) for radiocarbon dating

The bone collagen extraction procedure applied here is based on the Longin^18^ method and similar to the protocol described by Brock et al.^19^. Bone samples were demineralised in 0.2 M hydrochloric acid (HCl) for several days (mechanical and visual checks) during which the acid was renewed several times. Samples were rinsed three times with Milli-Q water, submerged in 0.1 M sodium hydroxide (NaOH) for 20 min (if discolouration appeared, new NaOH was added for another 20 min), rinsed three times with Milli-Q water, then submerged in 0.1 M HCl for 10 min, followed by three Milli-Q water rinses. Samples were gelatinised in weak (pH 3) HCl at 90 °C until dissolution, filtered using glass filter units (mesh size 10–20 μm), frozen using liquid nitrogen and lyophilised in clean (baked out) vials.

### *Combustion, graphitisation and *^*14*^*C measurements*

Collagen samples were connected to the CO_2_ extraction line of the radiocarbon laboratory at the Muséum national d’Histoire naturelle. After adding 900 mbar pure O_2_, samples were combusted at 900 °C for 10–20 min in the presence of a baked out silver strip (10 mg) to remove contaminants, and cleaned on the CO_2_ extraction line (water trap, NOx oven fitted with copper and silver fibre wool) and volume calculated. The vacuum on the line could reach 10^−6^ mbar. The CO_2_ gas sample was transferred to a semi-automated H_2_ reduction line using iron as a catalyst, where the vacuum could reach 10^−7^ mbar. Samples were run alongside standards (oxalic acid and phthalic acid). Graphite targets were dated using the ECHoMICADAS AMS at Gif-sur-Yvette (France). Data reduction was performed by BATS software (version 47)^20^. The first few scans were discarded to eliminate possible contamination of the target with ambient atmosphere between target pressing and AMS measurement. Radiocarbon ages were calculated from F^14^C^21^, which is corrected for blank and isotopic fractionation for the samples and only isotopic fractionation for blank values. Measurement parameters such as ^12^C current and ^13^CH current were checked. Time, current and isobar corrections were made prior to validation. Normalisation, correction for fractionation and background corrections were applied for each individual run by measuring the oxalic acid II NIST standard, its ^13^C/^12^C ratio and the chemical blanks. The standard deviation of the blanks is generally less than 10% but an overestimated standard deviation of 30% is imposed to the blank value, in order to take into account a potential variability of the contaminant which could be added during the sample preparation analysed.

### Collagen glue removal from Palaeolithic objects for ZooMS analysis

Two variations of the standard ZooMS acid extraction protocol^22^ were tested to see if the contaminating glue signature could be removed or at least reduced in the Palaeolithic bone objects. The first involved a double demineralisation/double gelatinisation step, in which samples of bone powder (20–40 mg) were placed in 200 μL of 0.6 M HCl at room temperature for 1 h. The acid was removed and the samples were rinsed three times with 200 μL of 50 mM ammonium bicarbonate (AmBic, pH 8) and gelatinised in 100 μL of AmBic for 30 min at 65 °C. The AmBic was transferred to a new tube, and a second round of demineralisation was performed with the addition of 200 μL of 0.6 M HCl for 2 h at room temperature. The acid was removed and the samples were rinsed three times with 200 μL AmBic, and gelatinised for 1 h at 65 °C in 100 μL of AmBic. Both the first and second gelatinisation solutions were digested with 0.4 μg/μL trypsin and incubated at 37 °C overnight. The trypsin was then stopped by acidifying the samples to 0.1% trifluoroacetic acid (TFA), and the samples were desalted using C18 zip tips (Pierce™). Samples were analysed in duplicate or triplicate with 1 μL of sample mixed with 1 μL of matrix solution [α-cyano-4-hydroxycinnamic acid (CHCA)] on a Bruker ground steel MALDI plate and analysed using a Bruker Ultraflex III Matrix Assisted Laser Desorption Ionisation Time of Flight Mass Spectrometer (MALDI-TOF-MS). Resulting spectra were averaged and analysed using mMass software^23^ and compared against a database of known species^24–27^.

The second variation involved a double gelatinisation step, in which bone powder samples (20–40 mg) were placed in 200 μL of AmBic at room temperature for 8 h on a shaker or with intermittent vortexing, then gelatinised for 30 min at 65 °C. The AmBic was transferred to a new tube, and 200 μL of 0.6 M HCl was added to the bone powder and left to demineralise for 2 h at room temperature. The acid was removed and the samples were rinsed three times with AmBic, then gelatinised in 100 μL of AmBic at 65 °C for 1 h. Both the first and second gelatinisation solutions were then digested, desalted, spotted and analysed as outlined above. An overview of how the Palaeolithic samples were treated is visible in Figure S2.

### ZooMS analysis of “cleaned” experimental bone and Palaeolithic objects

Six of the 18 experimentally contaminated bone samples that were “cleaned” using the two radiocarbon collagen removal processes had sufficient collagen remaining for ZooMS after the stable isotope and radiocarbon analyses (samples MUSE20026.40/41_ABA, MUSE20026.41, MUSE20026.44/45_ABA, MUSE20026.48, MUSE20026.49, and MUSE20026.48/49_ABA). For the “cleaned” Palaeolithic objects, only two samples (MUSE21164 and MUSE21169) had sufficient collagen remaining for ZooMS after the stable isotope and radiocarbon analyses. For all samples, approximately 0.5 mg of collagen was resuspended in 50 μL of AmBic. The samples were desalted, spotted and analysed as outlined above.

### FTIR-ATR for collagen glue detection

Bone powder (1 mg) was analysed by Fourier Transform Infrared Spectroscopy in Attenuated Total Reflectance mode (FTIR-ATR) by pressing the powder between the surface of a diamond crystal using a single reflection ATR-Golden Gate accessory (Specac) on a Vertex 70 spectrometer (Bruker) at the Musée de l’homme, Paris, France. Spectra were collected with a spectral resolution of 4 cm^−1^ for 32 scans in the range of 4000–370 cm^−1^. The anvil pressure on the ATR crystal was adjusted to obtain a raw absorbance of 0.5 for the ν_3_PO_4_ band around 1015 cm^−1^ and spectra of the bone powder samples were background corrected^28^. The anvil pressure adjustment using the phosphate peak was not possible for the collagen glues.

## Results and discussion

The atomic C:N ratios of the archaeological bone samples (Table 1) fall between 2.9 and 3.6^29^ (3.21 and 3.58), showing that the collagen is preserved well enough to produce a reliable ^14^C date, while the collagen yields (0.4% and 11.6%) should be between 1 and 22wt%^30^. However, five samples (MUSE21160, MUSE21161, MUSE21165, MUSE21168 and MUSE21171) produced insufficient collagen to perform both radiocarbon dating as well as IRMS. Two of these samples also had collagen yield below 1% (MUSE21160 (0.4%) and MUSE21171 (0.8%). IRMS specifics are available in the supplementary information.

**Table 1:** ZooMS results, collagen yields, atomic C:N ratios and radiocarbon ages from the 13 Palaeolithic bone objects.

Sample label	Year of excavation	Echo n°	Lab number	Species ID based on ZooMS	Collagen yield (%)	Atomic C:N ratio	^14^C age BP (yr)	±	Remark
630 IST II 1931	1931	4612.1.1	MUSE21159	sperm whale/*Bos*	7,2	3,21	12 850	60	
634 IST 77,162 B18	Unknown	4648.1.1	MUSE21160	sperm whale/*Bos*	0,4	*	–		GIS
637 IST E 77,159 D10	1914	4649.1.1	MUSE21161	sperm whale/*Bos*	2,1	*	–		GIS
749 IST I + II 1930	1930	4605.1.1	MUSE21162	fin whale/*Bos*	8,5	*	10 190	50	
780 IST I 1930	1930	4606.1.1	MUSE21163	sperm whale/*Bos*	2,5	*	4200	30	
2 IST EW 1920	1920	4613.1.1	MUSE21164	*Bos* with whale	9,3	3,58	7020	30	
7 IST SI 1929	1929	4650.1.1	MUSE21165	*Bos* with whale	3,8	*	–		GIS
10 IST II 1931	1931	4607.1.1	MUSE21166	sperm whale/*Bos*	2,0	*	13 540	70	
14 IST II 1932	1932	4608.1.1	MUSE21167	sperm whale/*Bos*	4,9		14 990	80	
19 IST SI 1929	1929	4651.1.1	MUSE21168	*Bos* with whale	2,1	*	–		GIS
38 IST II 1934	1934	4609.1.1	MUSE21169	sperm whale/*Bos*	11,6	3,28	14 130	70	
39 IST II 1937	1937	4614.1.1	MUSE21170	fin whale/*Bos*	5,0	3,27	13 830	60	
341 IST SI 1929	1929	4652.1.1	MUSE21171	*Bos* with reindeer	0,8	*	5660	140	GIS^1^

*Not enough material was available for IRMS. Several samples were too small to be analysed in graphite form and were analysed in gas form (GIS mode) on the ECHoMICADAS AMS.

^1^Sample mass (58 µgC) (more information see Table S2).

### Collagen glue removal for radiocarbon dating of experimental and archaeological bone

**Figure 2:**
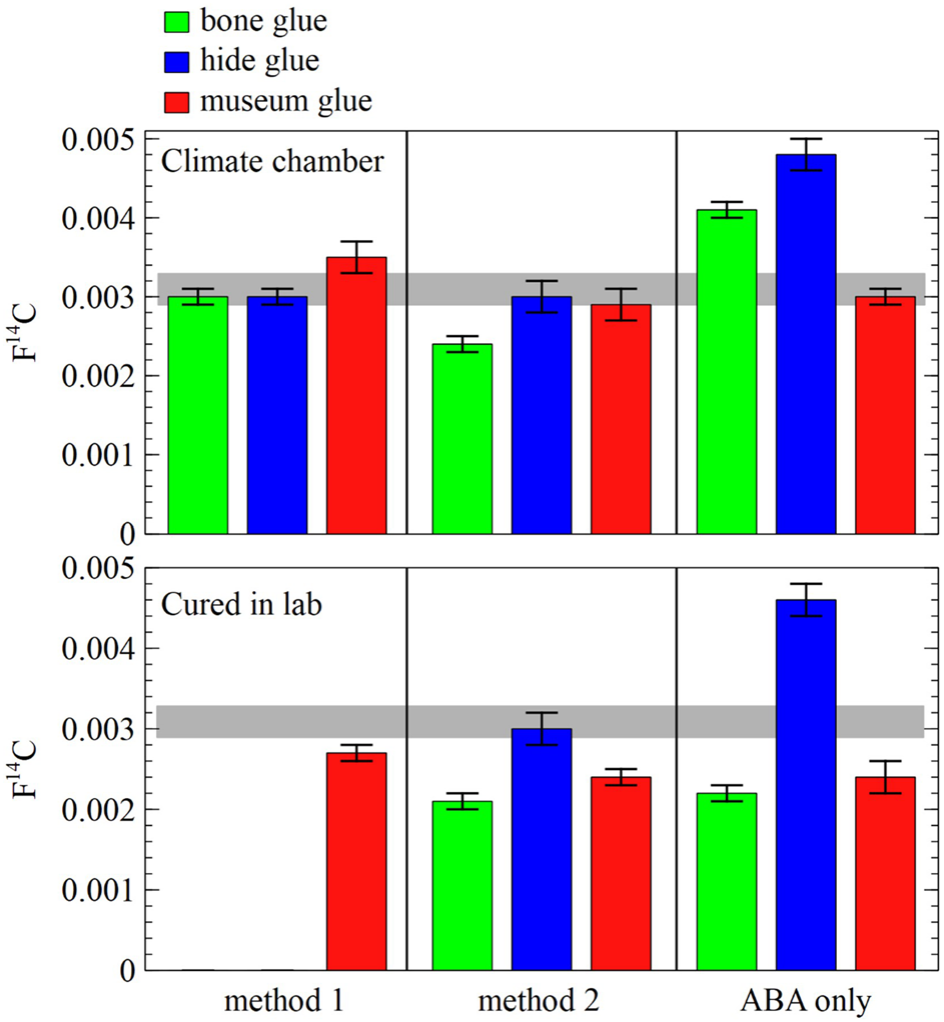
Radiocarbon results testing the three different cleaning methods and three different collagen glues, the grey bands marks the Hollis bone blank (FmC = 0.0031 ± 0.0002 (n = 219)^16^). Two samples are missing (cured in lab, method 1) due to cracking of the glass vials during freezing with liquid nitrogen.

Bone blanks that were consolidated with one of the three collagen glues were either cured in the laboratory for 28 days or artificially aged in a climate chamber for 28 days prior to subjecting them to cleaning methods and radiocarbon dating (Table S1). The results of two samples (MUSE20026.38 and MUSE20026.42) are missing because the glass vials cracked during freezing with liquid nitrogen. Although each cleaning method gave statistically different ^14^C ages (see Table S1), indicating none of them statistically worked better than another, the average ^14^C ages per cleaning method emerge in age in the following order: method 2 > method 1 > ABA only. Overall, cleaning method 2 gave the oldest blank ^14^C ages regardless of the glue type contamination (Fig. 2, see also Table S1). It appears that the museum glue was successfully removed regardless of which cleaning method was employed, although this was not the case for the bone and hide/skin glue, which were systematically better removed with method 2 for samples from the climate chamber. The results are less clear for samples that were cured in the laboratory. Both method 2 and the ABA-only method gave excellent results for bone glue and museum glue contaminated samples, while the hide/skin glue samples still contained small amounts of glue. Six of the 18 experimentally consolidated samples had sufficient collagen remaining after the other analyses to perform ZooMS. Only elephant/mammoth (which are indistinguishable using ZooMS) peptide markers were identified in all samples except for one, MUSE20026.44/45_ABA, which only had minimal residue remaining after the other analyses and failed to provide an ID. These results also suggest that all contaminating glue had been removed by the cleaning method used.

These results are much better than those from Takahashi et al.^13^, which is surprising as they only left their samples to cure for three days. However, they only treated their samples with a 20-min HCl wash, followed directly by gelatinisation. A large difference with their extraction protocol would thus be the inclusion of the NaOH wash and subsequent HCl step, which are currently normal practice in radiocarbon laboratories. As such, it appears that even the regular ABA treatment is able to remove sufficient, if not all, of the collagen glue from these experimental samples. Since it is impossible to differentiate between bone and hide/skin glue in the archaeological samples, cleaning method 2 was selected to treat the 13 samples from the Palaeolithic bone objects, as this method gave the most optimal radiocarbon results. Five samples produced very small amounts of collagen and subsequently such small amounts of CO_2_ that they had to be radiocarbon dated in GIS mode on the ECHoMICADAS AMS. Unfortunately, four of these samples failed the criteria required for GIS samples (Table 1).

### Collagen glue identification and removal using ZooMS

**Figure 3:**
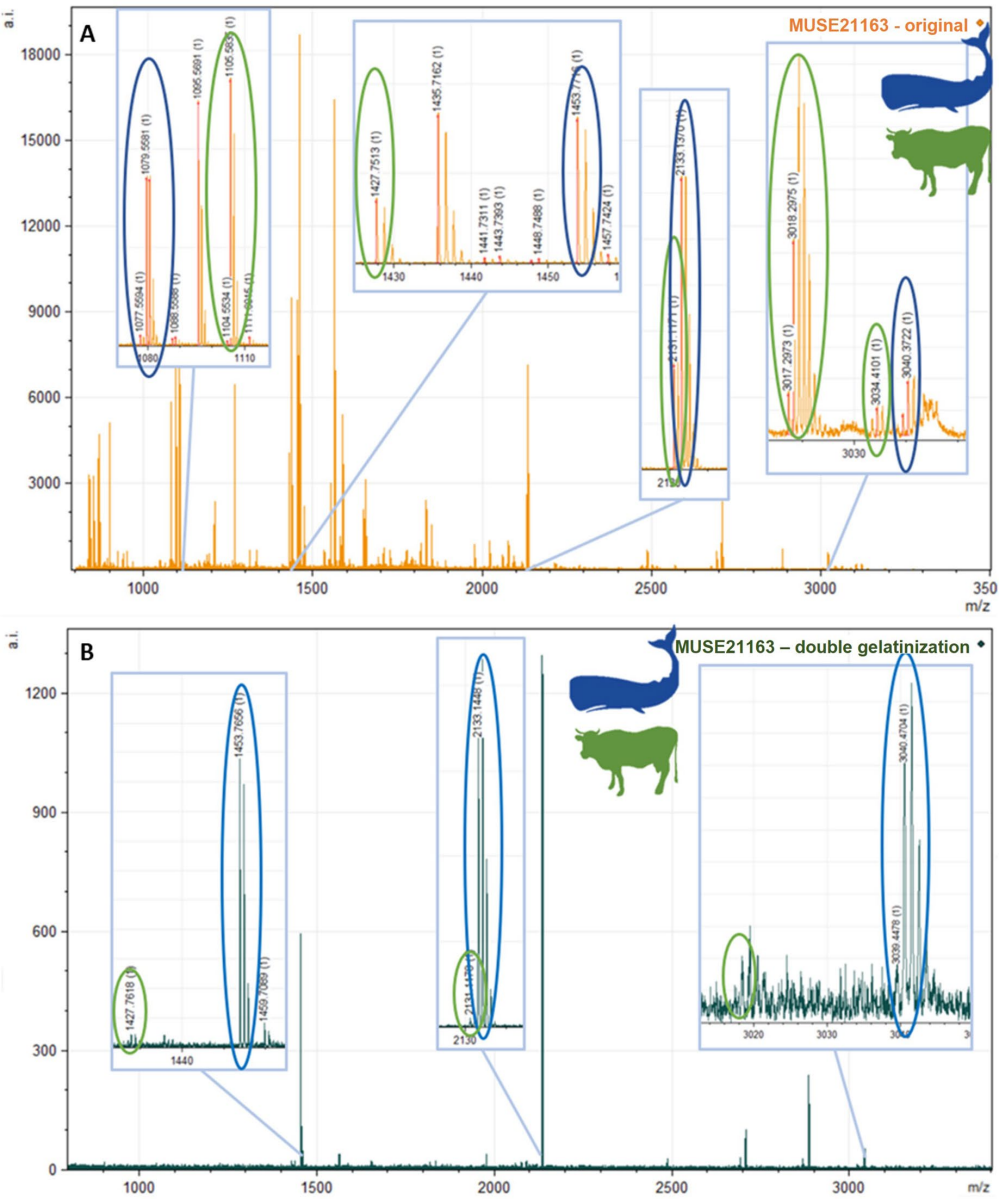
(**A**) ZooMS spectra showing peaks associated with the presence of both cattle and (sperm) whale peptide characteristics. (**B**) Spectra after the double gelatinisation treatment showing diminished cattle peaks.

**Figure 4:**
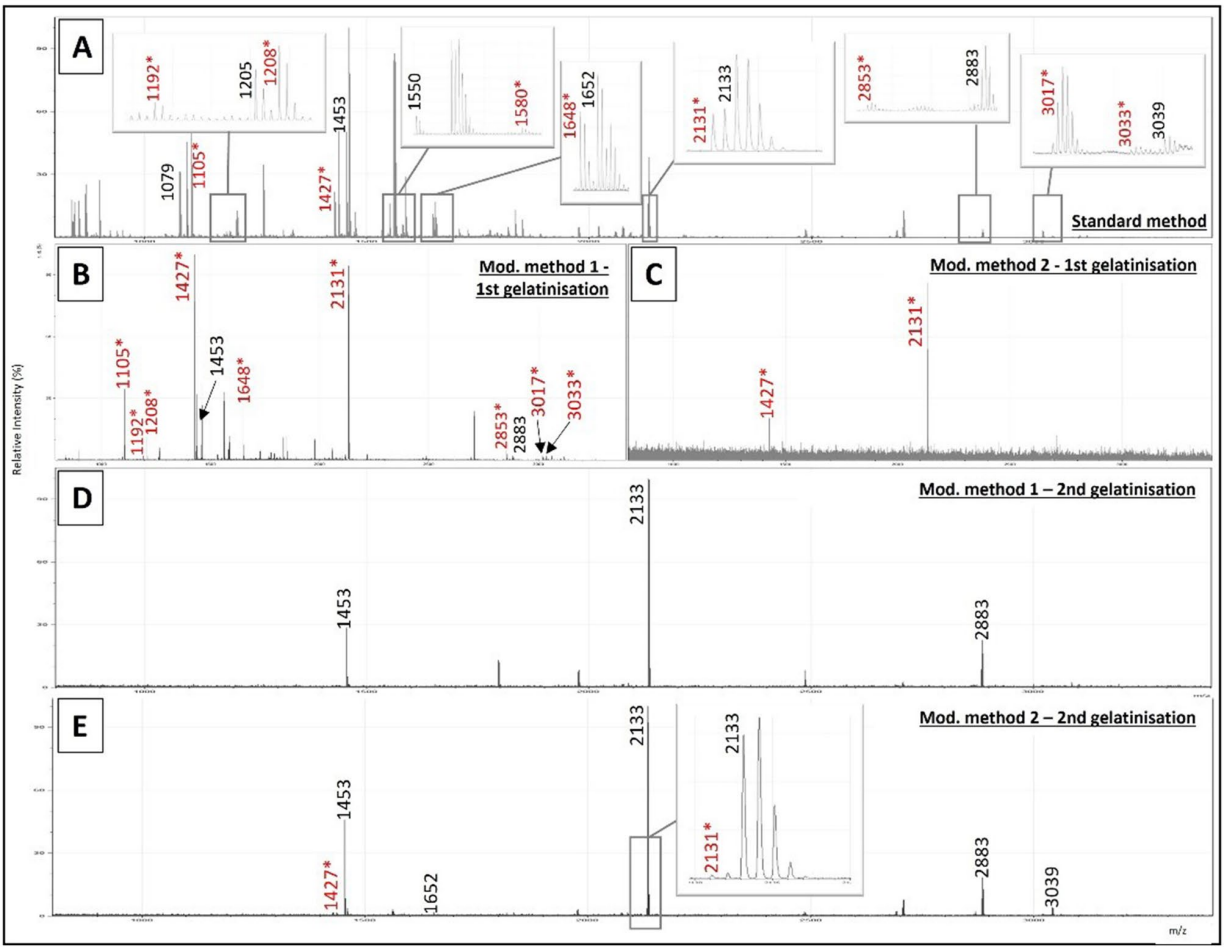
Spectra for all ZooMS extraction methods for sample MUSE21163. (**A**) Standard ZooMS acid extraction method showing both cattle and whale collagen peptide markers; (**B**) first gelatinisation from Modified Method 1 (double acid extraction) showing mostly cattle markers; (**C**) first gelatinisation from Modified Method 2 (double gelatinisation extraction) showing only cattle markers; (**D**) second gelatinisation from Modified Method 1 with whale peaks, however key markers to distinguish between species are absent due to high degradation; (**E**) second gelatinisation from Modified Method 2 (double gelatinisation extraction). Markers in red with * indicate cattle markers, while those in black are whale markers. In general, Modified Method 2 provided the best results, with substantially reduced cattle markers and sufficient whale markers to make high level (usually species) taxonomic identifications (inset E). Intensity has been normalised for comparative purposes.

The Palaeolithic objects were initially expected to be whale bone, however ZooMS revealed the presence of collagen peptide markers characteristic of cattle, and upon closer inspection, a mix of markers characteristic of both whale and cattle. This led to the conclusion that a consolidant in the form of cattle collagen glue had possibly been applied to the objects (Fig. 3A). Cattle (*Bos*) markers were identified at m/z 1105, 1427, 2131, 3017, 3033, while whale peaks were identified at m/z 1079, 1453, 2133, 2883, 3039 (Table 2)^24–27^. While animal collagen glue has previously been identified in artworks using MALDI-TOF-MS^31–33^, this is the first time it has been identified in archaeological remains using ZooMS, illustrating again the incredible versatility of this technique. In order to verify this and identify the authentic animal species used for the bone objects, the two ZooMS extraction methods were executed, under the assumption that contaminating collagen glue is not, or is at least less, bound to the original collagen molecule and could be removed. The first method applying double demineralisation/gelatinisation caused too much damage to the contaminating and endogenous collagen, resulting in very poor spectra. The second method using double gelatinisation resulted in a diminished cattle peptide fingerprint, while the whale peptide markers were generally more clearly defined (Fig. 3B), confirming that these were originally whale bone objects that had been consolidated with collagen-based cattle glue. The resulting spectra contained far fewer peaks and at a lower intensity than the original, which was expected as the initial gelatinisation step would have also removed some of the soluble whale bone collagen, and the subsequent acid demineralisation likely caused further collagen loss. However, with the double gelatinisation method, sufficient endogenous collagen remained to identify the species of animal whose bone was used for the production of the bone objects. An overview of these comparative spectra from all these different methods from sample MUSE21163 is shown in Fig. 4.

**Table 2:** Collagen peptide markers used to make the ZooMS identifications of the Palaeolithic samples after undergoing standard acid extraction and/or the modified double gelatinisation method to remove the contaminating cattle-collagen glue.

Original sample number	Lab number	Extraction method	ZooMS ID	Collagen peptide markers											
				P1	A1	A2	B	C	P2	D	E	F1	F2	G1	G2
630 IST II 1931	MUSE21159	Standard extraction	Bos with whale	1079?/**1105**	**1192**	**1208**	1453?/**1427**	**1580**	**1648**	**2131**	–	**2853**	–	**3017**	–
		Double gelatinisation	Sperm whale/Bos	1079/**1105**	–	1205/**1208**	1453/**1427**	1550	1652/**1648**	2133/**2131**	–	2883/**2853**	–	**3017**	3039/**3033**
634 IST 77,162 B18	MUSE21160	Standard extraction	Bos with whale	1079?/**1105**	–	–	1453/**1427**	–	**1648**	**2131**	–	2883/**2853**	–	**3017**	–
		Double gelatinisation	Sperm whale/Bos	1079/**1105**	–	**1208**	1453/**1427**	1550/**1580**	1652/**1648**	2133/**2131**	–	2883/**2853**	–	**3017**	3033
637 IST 77,159 D10	MUSE21161	Standard extraction	Probable Bos	**1105**	–	**1208**	1453?/**1427**	–	**1648**	**2131**	–	**2853?**	–	–	–
		Double gelatinisation	Sperm whale/Bos	1079/**1105**	–	**1208**	1453/**1427**	1550/**1580**	1652/**1648**	2133/**2131**	–	2883/**2853**	–	**3017**	3033
749 IST I + II 1930	MUSE21162	Standard extraction	Bos with whale	1079?/**1105**	**1192**	**1208**	**1427**	–	**1648**	**2131**	–	2883?/**2853**	–	**3017**	–
		Double gelatinisation	Fin whale/Bos	1079	–	–	1453/**1427**	–	1652	2135/**2131**	–	2883/**2853**	–	–	3023/**3033?**
780 IST I 1930	MUSE21163	Standard extraction	Sperm whale/Bos	1079/**1105**	1189/**1192**	1205/**1208**	1453/**1427**	1550/**1580**	1652/**1648**	2133/**2131**	–	2883/**2853**	–	**3017**	3039/**3033**
		Double gelatinisation	Sperm whale/Bos	–	–	–	1453/**1427**	–	1652	2133/**2131**	–	2883	–	**3017**	3039
2 IST EW 1920	MUSE21164	Double gelatinisation	Bos with whale	1079/**1105**	**1192**	1205/**1208**	1453/**1427**	**1580**	**1648**	**2131**	–	2883/**2853**	–	3023?/**3017**	3039?/**3033**
7 IST SI 1929	MUSE21165	Double gelatinisation	Bos with whale	1079/**1105**	**1192**	**1208**	1453/**1427**	**1580**	**1648**	**2131**	2766/**2792**	2883/**2853**	–	**3017**	–
10 IST 11 1931	MUSE21166	Double gelatinisation	Sperm whale/Bos	1079/**1105**	–	1205/**1208**	1453/**1427**	1550	1652/**1648**	2133/**2131**	–	2883/**2853**	–	**3017**	3039/**3033**
14 IST 11 1932	MUSE21167	Standard extraction	Probable Bos	**1105**	–	**1208**	**1427**	–	**1648**	**2131**	–	–	–	–	–
		Double gelatinisation	Sperm whale/Bos	1079/**1105**	–	1205/**1208**	1453/**1427**	1550/**1580**	1652/**1648**	2133/**2131**	2766?	2883/**2853**	–	3023/**3017**	3039/**3033**
19 IST SI 1929	MUSE21168	Double gelatinisation	Bos with whale	1079/**1105**	**1192**	**1208**	1453/**1427**	**1580**	**1648**	**2131**	2766/**2792**	2883/**2853**	**2869?**	**3017**	3033
38 IST 11 1934	MUSE21169	Standard extraction	Bos with whale	1079?/**1105**	**1192**	**1208**	1453?/**1427**	**1580**	**1648**	**2131**	–	**2853**	–	–	–
		Double gelatinisation	Sperm whale/Bos	1079	–	1205	1453/**1427?**	1550	1652	2133/**2131**	–	2883/**2853**	**2869**	3023/**3017**	3039/**3033**
39 IST 11 1937	MUSE21170	Standard extraction	Bos with whale	1079?/**1105**	**1192?**	**1208**	1453?/**1427**	–	**1648**	**2131**	–	**2853**	–	–	–
		Double gelatinisation	Fin whale/Bos	1079/**1105**	–	1205	1453/**1427**	–	1652/**1648**	2135/**2131**		2883/**2853**	**2869**	**3017**	3023/**3033**
341 IST SI 1929	MUSE21171	Double gelatinisation	Bos with reindeer	1105*	1150?/**1192**	1166?/**1208**	1427*	1580*	1648*	2131*	2792*	2883/**2853**	2899	**3017**	3093/**3033**

Peptide markers in bold are the cattle markers. ? indicates that the marker was likely present however it was below the m/z threshold or in an area of poor resolution; – indicates no marker was present; * indicates the same marker is used for both the species of origin and the contaminating glue species.

The majority of the samples (n = 7) were identified as sperm whale, along with two fin whales, three samples which could only be identified as cetaceans, and one reindeer (the antler sample analysed for comparison: Table 1). While the cattle peaks were significantly reduced and occasionally appeared to be gone using the double gelatinisation (Fig. 4), it is impossible to say with absolute certainty that the cattle signal was ever completely absent, which could have implications for the radiocarbon dating of these samples.

**Figure 5:**
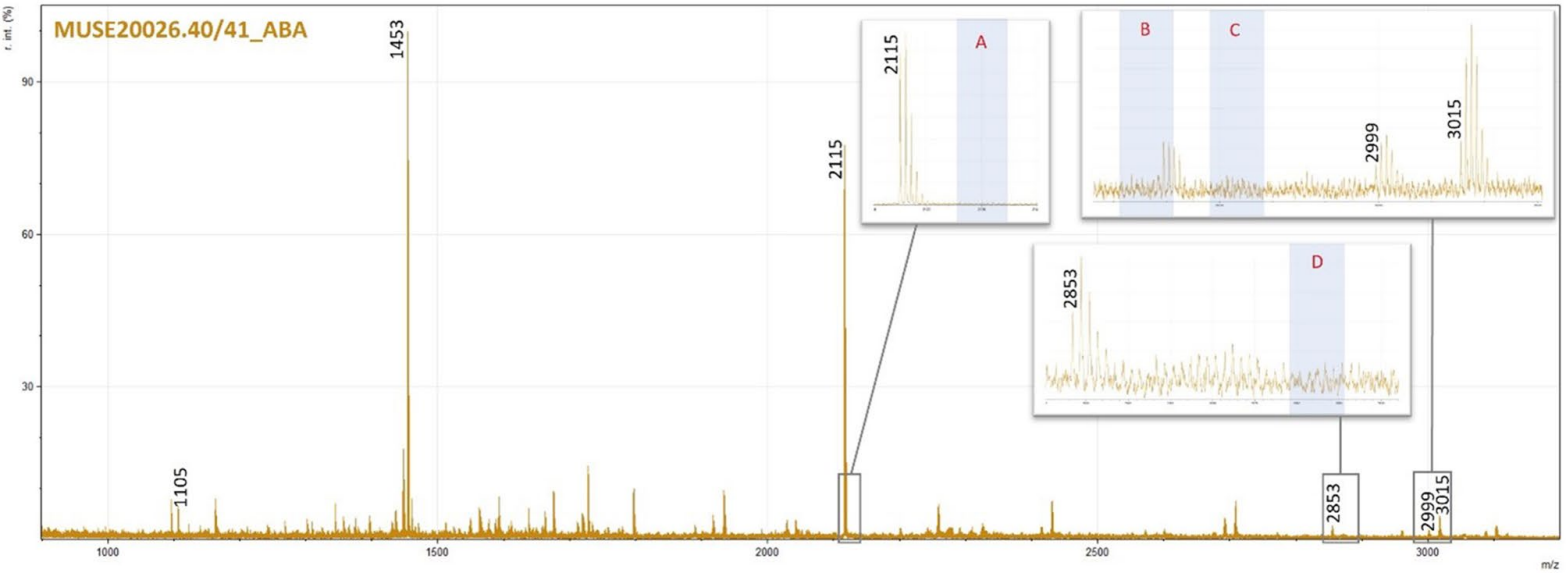
Hollis post clean: ZooMS spectra for contaminated Hollis bone sample MUSE20026.40/41_ABA after consolidant removal using the standard ABA collagen extraction protocol. Labelled peaks show those used to identify the bone as elephant/mammoth. Shaded bands in the inset images indicate where rabbit markers would be expected if present (A—m/z 2129, B—m/z 2957, C—m/z 2973, D—m/z 2883).

**Figure 6:**
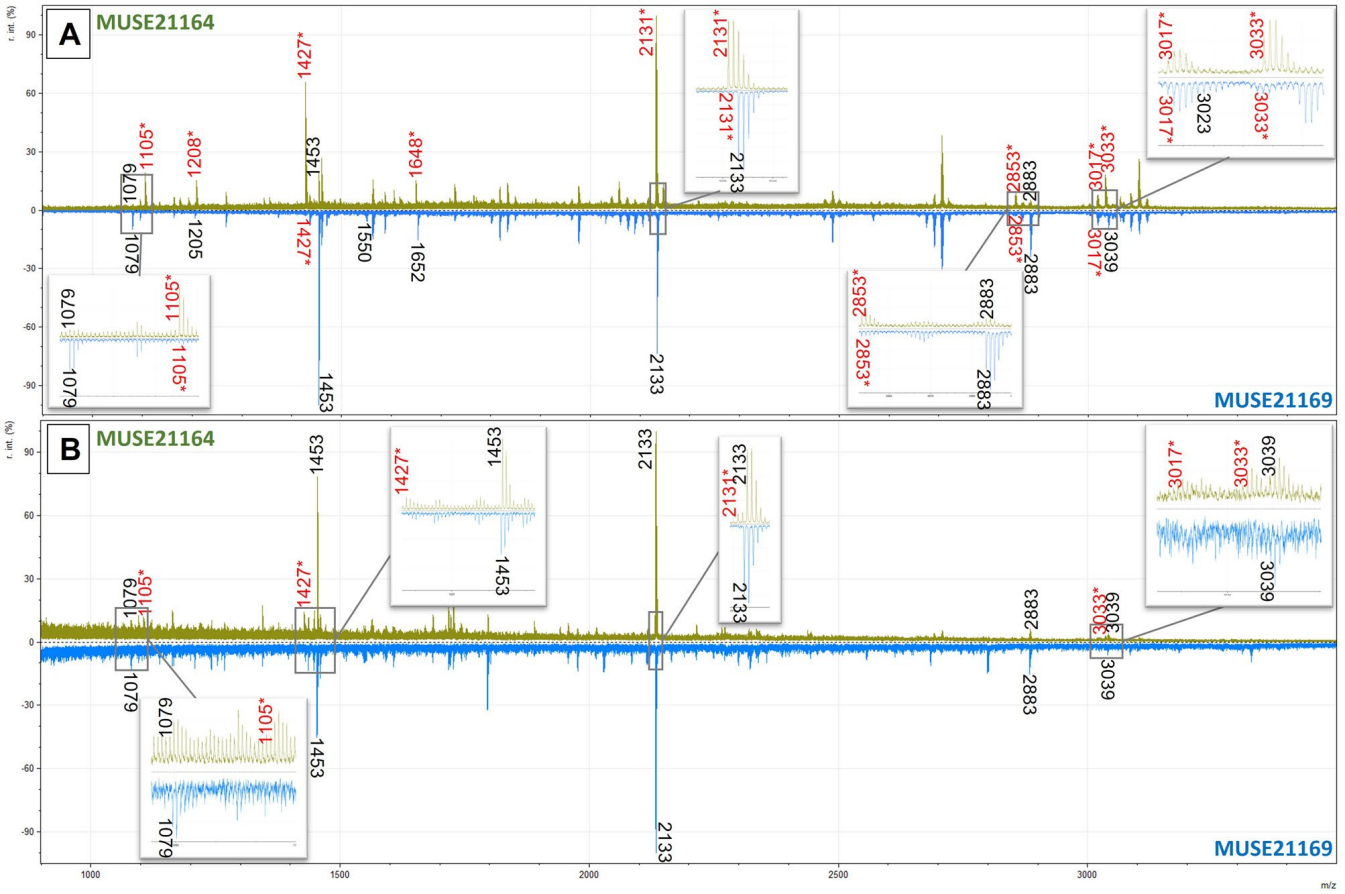
ZooMS spectra for samples MUSE21164 (top green spectra) and MUSE21169 (bottom blue spectra) after undergoing (**A**) standard acid extraction and (**B**) the radiocarbon cleaning protocol to try to remove the contaminating consolidant. Peak labels in red with * indicate cattle markers while those in black are whale markers. Fewer cattle markers can be identified after the radiocarbon cleaning method 2 + ABA for both samples (inset B), and the cattle markers that are present are significantly reduced in comparison to the whale markers. However, the presence of cattle markers means that not all glue has been removed and this contamination is problematic for radiocarbon dating these samples. Intensity has been normalised for comparative purposes.

Four of the six experimentally contaminated mammoth bone samples analysed with ZooMS after undergoing the cleaning protocols were identified as elephant/mammoth with no indication of contaminating peptides from the glues (rabbit peptides in the case of the modern hide and bone glues) (Fig. 5 and Table 3). Two of the samples returned very poor spectra with no identifications possible using ZooMS. Although reduced, cattle collagen glue contamination could still be seen in the ZooMS spectra for the two Palaeolithic samples (MUSE21164, MUSE2169) that underwent the radiocarbon cleaning protocol (Fig. 6), with MUSE21164 providing a radiocarbon date significantly younger than expected for the site context. These ZooMS results, along with the younger than expected radiocarbon dates, suggest that while the cleaning protocol was able to remove the glue in the experimental samples, it was not sufficient for removing collagen glues which have been on objects for several decades, indicating the collagen glue peptides have likely cross-linked with the endogenous collagen peptides^34^.

**Table 3:** Collagen peptide markers used to make the ZooMS identifications of the experimentally contaminated Hollis bone samples and two Palaeolithic samples (2 IST EW 1920 and 38 IST 11 1934) after undergoing different radiocarbon dating cleaning methods.

Original sample number	Lab number	Extraction method	ZooMS ID	Collagen peptide markers											
				P1	A1	A2	B	C	P2	D	E	F1	F2	G1	G2
			*Elephant/Mammoth*	*1105*	*1145*	*1161*	*1453*	*1579*	*1556*	*2115*	*2808*	*2853*	*2869*	*2999*	*3015*
			*European rabbit*	*1105*	*1221*	*1237*	*1453*	–	–	*2129*	–	*2883*	*2899*	*2957*	*2973*
Hollis bone	MUSE20026.41	RC cleaning method 1	Elephant/Mammoth	1105	–	–	1453	–	–	2115	–	–	–	–	3015?
Hollis bone	MUSE20026.40/41_ABA	RC cleaning method 2	Elephant/Mammoth	1105	–	1161	1453	–	–	2115	–	2853	–	2999	3015
Hollis bone	MUSE20026.48	RC cleaning method 1	Elephant/Mammoth	1105	1145?	1161	1453	–	–	2115	–	2853	–	2999	3015
Hollis bone	MUSE20026.49	RC cleaning method 1	Elephant/Mammoth	1105	–	–	1453	–	–	2115	–	2853	2869?	2999?	3015
Hollis bone	MUSE20026.48/49_ABA	RC cleaning method 2	Probable Elephant/Mammoth	1105	–	–	1453	–	–	2115	–	2853?	–	–	–
2 IST EW 1920	MUSE21164	RC cleaning method 2	Sperm whale/Bos	1079/**1105**	–	–	1453/**1427**	–	1652/**1648**	2133/**2131**	–	2883	–	3023?/**3017?**	3039/**3033**
38 IST 11 1934	MUSE21169	RC cleaning method 2	Sperm whale	1079	–	**1208**	1453	–	–	2133	–	2883	–	–	3039?

Hollis sample MUSE20026.44/45_ABA is not listed as it provided poor quality spectra resulting in no identification. The first two rows are previously published Elephant/Mammoth and European rabbit reference markers. Peptide markers in bold are the cattle-collagen glue markers. No unique markers (those not shared with the species of origin) were observed for the experimental rabbit glue in the Hollis bone samples. ? indicates that the marker was likely present however it was below the m/z threshold or in an area of poor resolution; –indicates no marker was present.

### Detecting collagen glue with FTIR-ATR

**Figure 7:**
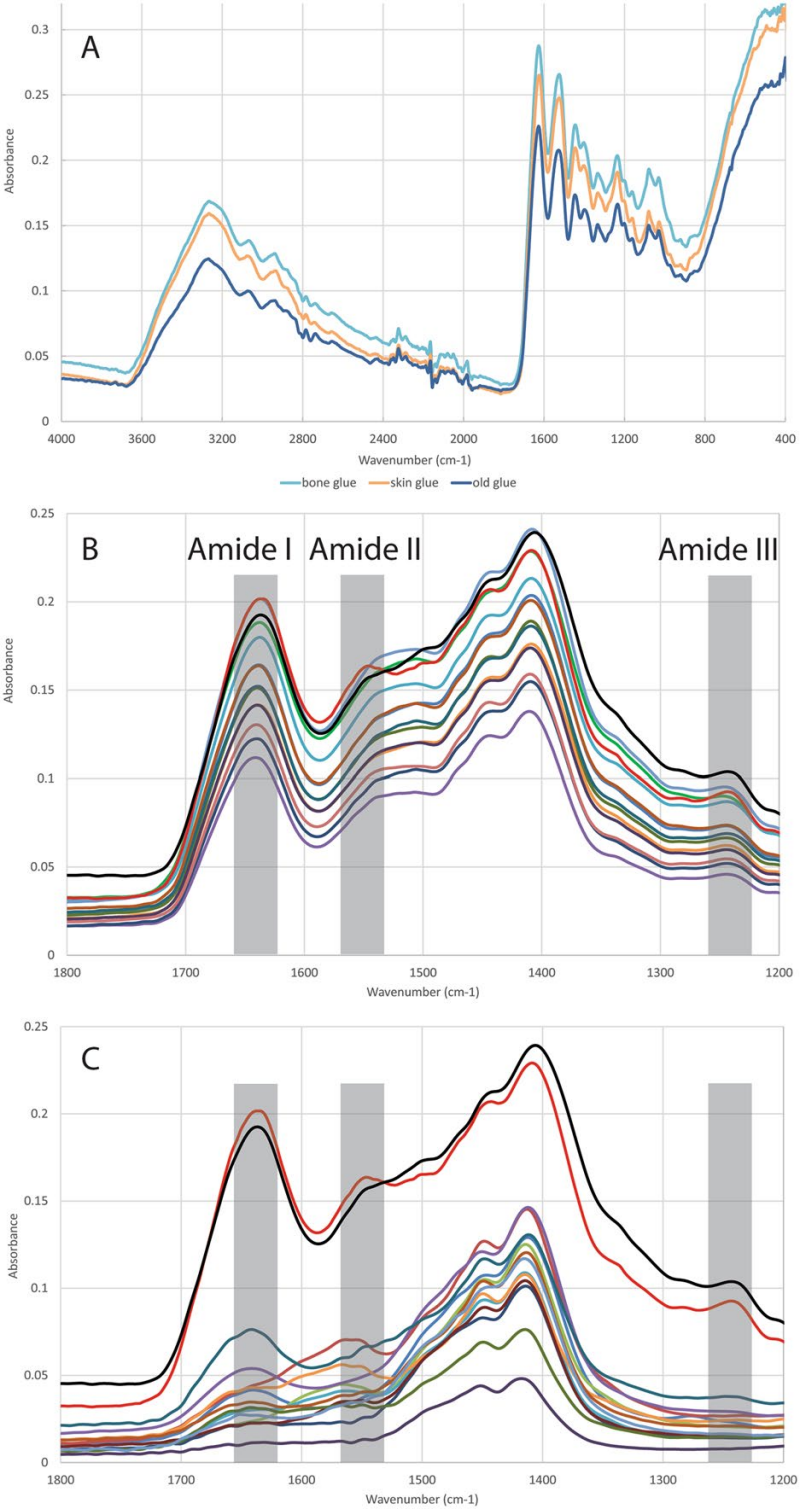
FTIR-ATR spectra of (**A**) the three collagen glues (4000–370 cm^−1^) (not adjusted with the phosphate peak), which produced the same spectra (**B**) a modern reference in red, a clean bone blank sample in black, and the collagen glue contaminated bone blank samples (1800–1200 cm^−1^) and (**C**) the spectra of the 13 Palaeolithic bone samples with a modern reference in red, clean bone blank sample in black (1800–1200 cm^−1^).

Due to similarities in the composition, the three collagen glues could not be distinguished in FTIR-ATR spectra (Fig. 7A). There was also no difference visible between uncontaminated samples and the samples consolidated with either of the three glues (Fig. 7B). There seemed to be a slight bulge around the Amide II peak, although this similarly appeared to be present in a clean bone blank sample but not in the modern reference spectrum. Some of the 13 Palaeolithic samples showed an increase in the Amide II peak around ~ 1575 cm^−1^, but not all of the samples (Fig. 7C). It is unusual that the Amide II peak (~ 1575 cm^−1^) would be stronger than the Amide I peak (~ 1650 cm^−1^). Considering that the collagen glues have peaks at all three Amide peaks, the increase around the Amide II peak is most likely not caused by the collagen glues. These peaks were similarly observed in other bone samples whose ZooMS spectra did not reveal any collagen glue, and are most likely the result of humic acids present in these bone samples^35^. FTIR-ATR is therefore not a suitable technique for the identification of collagen glue presence in archaeological bone. Nevertheless, the results did show that the Palaeolithic material is very poorly preserved, as the Amide I peak is considerably lower compared with the modern reference sample (Fig. 7C).

### Collagen glue removal and cross-linking

The Palaeolithic bone objects, which were recovered from Middle and Upper Magdalenian deposits, are expected to date between ca. 15 000 and 12 000 BP. Previous dating programs on material from the same layers yielded 21 radiocarbon dates ranging from 15 130 ± 110 BP to 12 185 ± 55 BP on terrestrial samples^36–38^. While five of the samples here produced ^14^C ages that are in keeping with this age range (MUSE21159, MUSE21166, MUSE21167, MUSE21169 and MUSE21170), other samples gave ^14^C ages that were younger (Table 1). ZooMS analysis of two of the samples, MUSE21164 and MUSE21169, after cleaning with method 2 also indicated that the collagen-based cattle glue had not been entirely removed as the spectra still contained a mixture of both cattle and whale peptide markers. The presence of remaining cattle glue in sample MUSE21169 indicates that this sample has not been cleaned sufficiently and that the ^14^C age, despite being in keeping with the radiocarbon ages of the deposit, is unreliable. It is therefore impossible to be absolutely certain that all collagen-based cattle glue was removed from any of these samples. At best, we can assign a minimal age to the bone objects that are in keeping with the expected age range obtained for this site. It is worth noting that marine (fish) collagen has a lower denaturation temperature than mammalian collagen due to the lower content of the amino acids proline and hydroxyproline^39^, which could mean that our cleaning method may have been more successful in the case of fish glue.

It seems most likely that the collagen glue has cross-linked to the endogenous collagen molecules in at least some of these bone objects. Cross-linked contamination can only be removed by breaking apart the collagen structure through hydrolysis, thereby releasing the contamination and breaking of the collagen into amino acids. In case the contamination is hydrophobic in nature (synthetic consolidants and humic acids), these can subsequently be removed by either XAD resin treatment^40,41^ or by radiocarbon dating only the amino acid hydroxyproline^42,43^. However, these methods are unlikely to provide a solution in the case of collagen glue contamination, as the glue will equally break down into its amino acids during the hydrolysis step, and it would be impossible to separate the amino acids originating from the endogenous collagen molecules and the glue collagen. These results also suggest that the artificial ageing in the climate chamber most likely did not induce cross-linking in the experimentally consolidated material, which could be related to either the type of consolidant (collagen based) or the duration of the artificial ageing. While artificial ageing is a good attempt to replicate archaeological material, it remains challenging to replicate archaeological samples that have been in contact with the collagen glue for decades, if not centuries. However, more testing can be done with different parameters (duration, temperature, pH) to improve the approach to induce cross-linking in such samples.

## Conclusion

The results presented here show that bone from old collections or old excavations should not blindly be subjected to radiocarbon dating, ancient DNA analysis, stable isotope analysis, palaeoproteomics or other chemical analyses without running some preliminary tests first. For such samples, we strongly recommend to first perform ZooMS in order to scan for the presence of collagen glue contamination as this can severely hamper the acquisition of reliable chemically derived information. ZooMS is a rapid and inexpensive technique that is becoming increasingly available^44^ and, as has been shown here, can be used to effectively detect collagen contamination. However, it must be kept in mind that if the animal of origin is the same as, or closely related to, the species from which the collagen glue was made, it will be impossible to distinguish between the two collagen sources. Similarly, spectra must be analysed carefully, with the intention of looking for contaminating peaks, as endogenous markers can be easily obscured by the peptides of the contaminating collagen glue using ZooMS. While FTIR-ATR proved ineffective for detecting collagen glue, it is useful to scan for the presence of any synthetic consolidants and obtain an estimate of the bone preservation and potential collagen yield.

There is currently no solution for reliable radiocarbon dating of samples contaminated with collagen glue. More research is needed to investigate both the scale of collagen glue contaminated samples in museums and archives, as well as ideas on how to successfully remove it. Future experiments could consist of a consolidation experiment designed to let samples naturally age in a museum environment for several years or to improve the artificial ageing method with various parameters.

We strongly advise against the continued use of animal-based glues, collagen (bone and hide) or otherwise, for conservation treatments of archaeological and palaeontological remains due to their close similarity to an artefact’s endogenous collagen, rendering it nearly impossible to remove adequately or with certainty. While this may seem ungrateful towards conservators in the past, this is certainly not the case. The archaeological and palaeontological remains that are currently at our disposal have only been preserved precisely due to these conservation specialists, without whose interference the material could have perished completely. Additionally, it would have been impossible to foresee how any conservation treatments would affect analytical work decades or hundreds of years later. Nevertheless, with the field of archaeological science rapidly expanding with new and more sensitive analytical techniques, more research is needed to better understand the potential effects that different conservation and consolidation materials and methods may have on archaeological material.

## Data availability

All data generated or analysed during this study are included in this published article. The raw maldi spectra and the FTIR-ATR files are available in the supplementary information files. The archaeological objects from Isturitz are from the Passemard and Saint-Périer collections, both curated at the Musée d’Archéologie Nationale in Saint-Germain-en-Laye. Permission for sampling was granted by Catherine Schwab, curator of the Palaeolithic Department of said museum.

### Supplementary Information

Supplementary Information 1.

Supplementary Information 2.

Supplementary Information 3.
