# Supplementary material for: Identification and tentative removal of collagen glue in Palaeolithic worked bone objects: implications for ZooMS and radiocarbon dating
Source: Sci Rep. 2023 Dec 13;13:22119. doi: 10.1038/s41598-023-49242-7 (PMC10719399; doi:10.1038/s41598-023-49242-7)
Supplement: Supplementary file 3 — Supplementary Information 3. [file 41598_2023_49242_MOESM3_ESM.docx]

Supplementary information


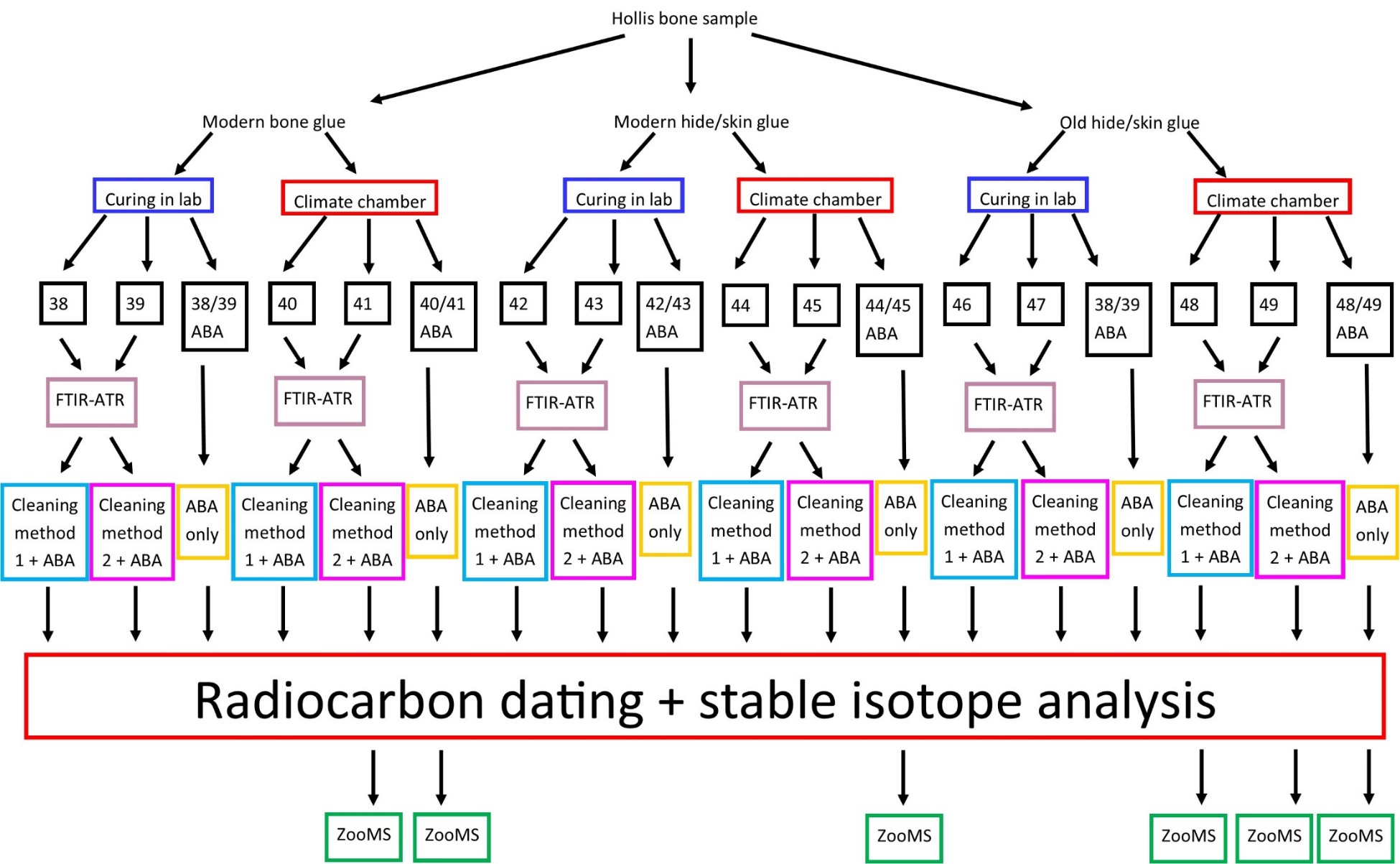


Figure S1. Flowchart illustrating how each subsample of Hollis bone blank was consolidated, aged in the lab or in the climate chamber, followed by FTIR-ATR analysis, and subjected to a cleaning method prior to being radiocarbon dated and analysed for stable isotopes. Six samples had collagen leftover that was analysed by ZooMS.


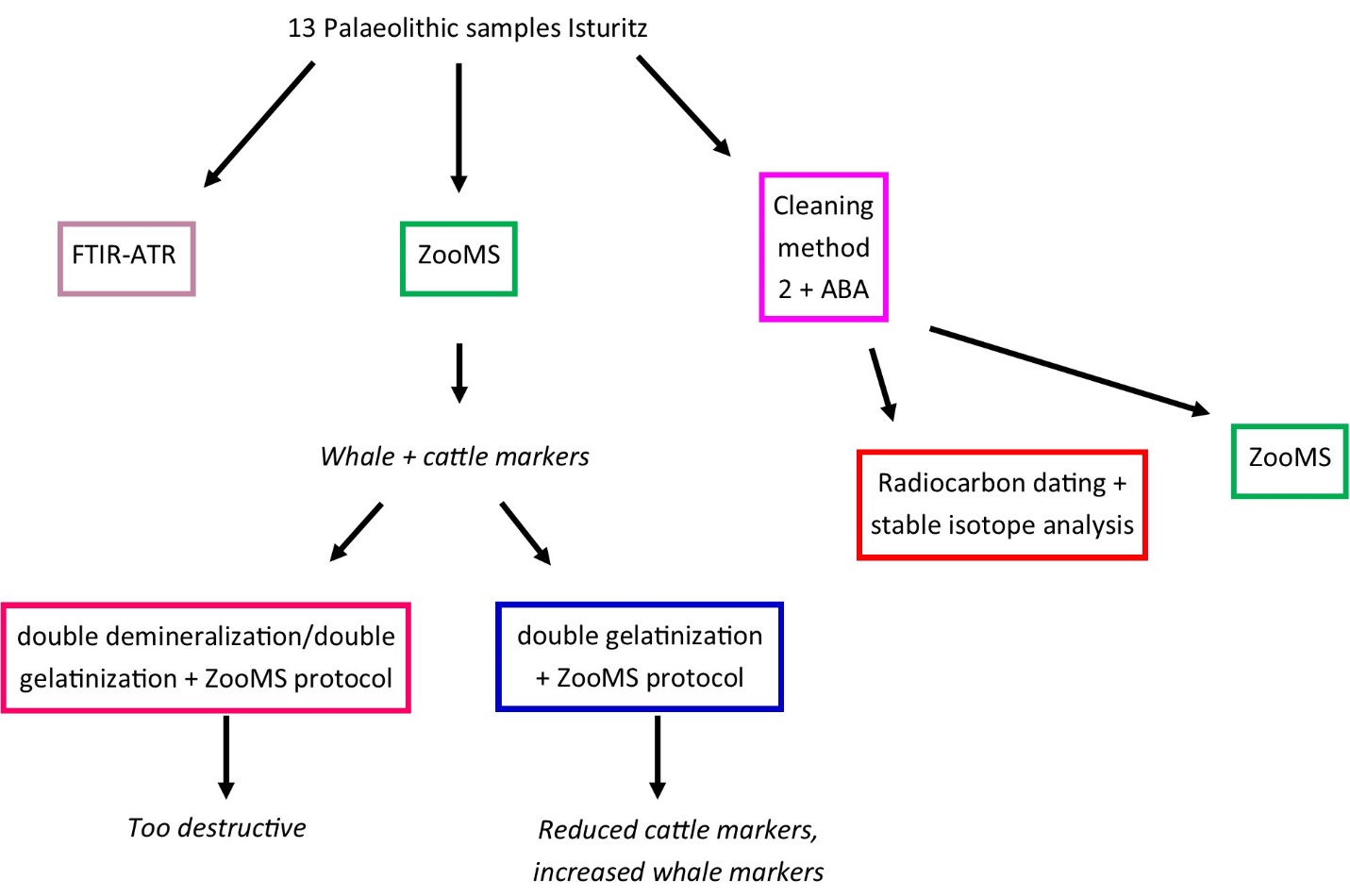


Figure S2. Flowchart illustrating how the 13 Palaeolithic samples from Isturitz were treated using various cleaning protocols for ZooMS and radiocarbon dating.

Table S1. Overview of the radiocarbon results from the consolidation experiment.

| **sample number** | **Echo n°** | **sample** | **glue type** | **cleaning method** | **collagen yield (%)** | **F14C** | **±** | **^14^C age yr BP** | **± (y)** | **remark** |
| --- | --- | --- | --- | --- | --- | --- | --- | --- | --- | --- |
| Blanks cured in the lab |  |  |  |  |  |  |  |  |  |  |
| MUSE20026.38 | - | Hollis mammoth | bone glue | Method 1 + ABA | - | - | - | - | - | vial cracked during freezing* |
| MUSE20026.39 | 3496.1.68 | Hollis mammoth | bone glue | Method 2 + ABA | 10.5 | 0.0021 | 0.0001 | 49 360 | 510 |  |
| MUSE20026.38/39_ABA | 3496.1.67 | Hollis mammoth | bone glue | ABA | - | 0.0022 | 0.0001 | 49 220 | 470 | vial cracked during freezing ^ |
| MUSE20026.42 | - | Hollis mammoth | hide glue | Method 1 + ABA | - | - | - | - | - | vial cracked during freezing * |
| MUSE20026.43 | 3496.1.69 | Hollis mammoth | hide glue | Method 2 + ABA | 13.4 | 0.0030 | 0.0002 | 46 640 | 380 |  |
| MUSE20026.42/43_ABA | 3496.1.72 | Hollis mammoth | hide glue | ABA | 13.2 | 0.0046 | 0.0002 | 43 320 | 300 |  |
| MUSE20026.46 | 3496.1.70 | Hollis mammoth | museum glue | Method 1 + ABA | - | 0.0027 | 0.0001 | 47 500 | 430 | vial cracked during freezing ^ |
| MUSE20026.47 | 3496.1.71 | Hollis mammoth | museum glue | Method 2 + ABA | 12.4 | 0.0024 | 0.0001 | 48 600 | 470 |  |
| MUSE20026.46/47_ABA | 3496.1.73 | Hollis mammoth | museum glue | ABA | 11.6 | 0.0024 | 0.0002 | 48 550 | 450 |  |
| Blanks aged in climate chamber |  |  |  |  |  |  |  |  |  |  |
| MUSE20026.40 | 3496.1.74 | Hollis mammoth | bone glue | Method 1 + ABA | 15 | 0.0030 | 0.0001 | 46 630 | 350 |  |
| MUSE20026.41 | 3496.1.75 | Hollis mammoth | bone glue | Method 2 + ABA | 15.3 | 0.0024 | 0.0001 | 48 310 | 440 |  |
| MUSE20026.40/41 | 3496.1.76 | Hollis mammoth | bone glue | ABA | 6.5 | 0.0041 | 0.0001 | 44 130 | 280 |  |
| MUSE20026.44 | 3496.1.77 | Hollis mammoth | hide glue | Method 1 + ABA | 17.3 | 0.0030 | 0.0001 | 46 780 | 360 |  |
| MUSE20026.45 | 3496.1.78 | Hollis mammoth | hide glue | Method 2 + ABA | 20.8 | 0.0030 | 0.0002 | 46 740 | 330 |  |
| MUSE20026.44/45 | 3496.1.79 | Hollis mammoth | hide glue | ABA | 16 | 0.0048 | 0.0002 | 42 930 | 250 |  |
| MUSE20026.48 | 3496.1.80 | Hollis mammoth | museum glue | Method 1 + ABA | 16.3 | 0.0035 | 0.0002 | 45 320 | 300 |  |
| MUSE20026.49 | 3496.1.81 | Hollis mammoth | museum glue | Method 2 + ABA | 14.6 | 0.0029 | 0.0002 | 47 000 | 360 |  |
| MUSE20026.48/49 | 3496.1.82 | Hollis mammoth | museum glue | ABA | 17.9 | 0.0030 | 0.0001 | 46 630 | 300 |  |
| Uncontaminated blanks |  |  |  |  |  |  |  |  |  |  |
| MUSE20026.15.2 | 3496.1.83 | Hollis mammoth |  | ABA | 15.5 | 0.0016 | 0.0001 | 51 650 | 530 |  |
| MUSE20026.25.2 | 3496.1.84 | Hollis mammoth |  | ABA | 21.3 | 0.0018 | 0.0001 | 50 970 | 560 |  |
| MUSE21003.15.1 | 3496.3.13 | Hollis mammoth |  | ABA | 17.5 | 0.0028 | 0.0001 | 47 090 | 350 |  |
| Collagen glues |  |  |  |  |  |  |  |  |  |  |
| MUSE21156 | 4580.1.1 | bone glue |  |  |  | 1.0150 | 0.0028 | -120 | 20 |  |
| MUSE21157 | 4581.1.1 | hide glue |  |  |  | 1.0353 | 0.0031 | -279 | 20 |  |
| MUSE21158 | 4582.1.1 | museum glue |  |  |  | 1.6392 | 0.0047 | -3970 | 20 |  |
|  |  |  |  |  |  |  |  |  |  |  |
|  |  |  |  | average method 1 + ABA | 46558 | 360 |  |  |  |  |
|  |  |  |  | average method 2+ ABA | 47775 | 415 |  |  |  |  |
|  |  |  |  | ABA only | 45797 | 342 |  |  |  | Χ^2^ (0.05) =5.99, T’ = 13.54  ^14^C ages are statistically different |
|  |  |  |  |  |  |  |  |  |  |  |
|  |  |  |  | method 1+ ABA + method 2+ABA | 47288 | 393 |  |  |  |  |
|  |  |  |  | ABA only | 45797 | 342 |  |  |  | Χ^2^ (0.05) =3.84, T’ = 8.19  ^14^C ages are statistically different |
|  |  |  |  |  |  |  |  |  |  |  |
|  |  |  |  | uncontaminated blanks | 49903 | 480 |  |  |  | Χ^2^ (0.05) =5.99, T’ = 67.15  ^14^C ages are statistically different |

*Collagen sample touched outside surface and could not be radiocarbon dated.

^Collagen was still intact but a reliable collagen yield could not be obtained.

Table S2. Radiocarbon results from the archaeological samples analysed in GIS mode. Samples need to have a mass > 10 µgC, a current > 4 µA and time of analysis > 5 minutes (300 sec). The correction has been made with a constant contamination correction of 0.545 ± 0.4 µgC and a ratio F^14^C of 0.57 ± 0.11.

| **Echo n°** | **label** | **F^14^C** | **error F^14^C** | **^14^C age** | **error age** | **current ^12^C (µA)** | **mass (µg C)** | **time of analysis (sec)** |
| --- | --- | --- | --- | --- | --- | --- | --- | --- |
| 4652.1.1 | MUSE21171 | 0.4942 | 0.0087 | 5 660 | 140 | 6.0 | 58 | 867.3 |

*Sample mass is below the smallest blank and standard mass measured (32 µgC).

IRMS

Collagen samples (320-380 μg) from the archaeological bone samples were weighed into tin capsules and analysed with a Thermo Scientific EA Flash 2000 coupled to a Delta V Advantage isotopic mass spectrometer. Isotopic values of all samples were measured relative to the laboratory standard alanine, which has a reproducibility of 0.3 wt% for N and 0.6 wt% for C. δ^13^C and δ^15^N values are reported relative to the VPDB and AIR, respectively. Analytical precision is ±0.2‰ for both δ^13^C and δ^15^N values.
